# Factors Influencing Functional Heterogeneity in Human Mucosa-Associated Invariant T Cells

**DOI:** 10.3389/fimmu.2018.01602

**Published:** 2018-07-10

**Authors:** Joana Dias, Caroline Boulouis, Michał J. Sobkowiak, Kerri G. Lal, Johanna Emgård, Marcus Buggert, Tiphaine Parrot, Jean-Baptiste Gorin, Edwin Leeansyah, Johan K. Sandberg

**Affiliations:** ^1^Center for Infectious Medicine, Department of Medicine, Karolinska Institutet, Karolinska University Hospital Huddinge, Stockholm, Sweden; ^2^U.S. Military HIV Research Program, Walter Reed Army Institute of Research, Silver Spring, MD, United States; ^3^Henry M. Jackson Foundation for the Advancement of Military Medicine, Bethesda, MD, United States; ^4^Program in Emerging Infectious Diseases, Duke-National University of Singapore Medical School, Singapore, Singapore

**Keywords:** mucosa-associated invariant T cells, MHC class I-related, CD56, cytokines, microbial immunity, antibacterial immunity, mucosal immunology

## Abstract

Mucosa-associated invariant T (MAIT) cells are unconventional innate-like T cells that recognize microbial riboflavin metabolites presented by the monomorphic MHC class I-related (MR1) molecule. Despite the high level of evolutionary conservation of MR1 and the limited diversity of known antigens, human MAIT cells and their responses may not be as homogeneous as previously thought. Here, we review recent findings indicating that MAIT cells display microbe-specific response patterns with multiple layers of heterogeneity. The natural killer cell receptor CD56 marks a MAIT cell subset with distinct response profile, and the T cell receptor β-chain diversity influences responsiveness at the single cell level. The MAIT cell tissue localization also influences their response profiles with higher IL-17 in tissue-resident MAIT cells. Furthermore, there is emerging evidence that the type of antigen-presenting cells, and innate cytokines produced by such cells, influence the quality of the ensuing MAIT cell response. On the microbial side, the expression patterns of MR1-presented antigenic and non-antigenic compounds, expression of other bioactive microbial products, and of innate pattern recognition ligands all influence downstream MAIT cell responses. These recent findings deepen our understanding of MAIT cell functional diversity and adaptation to the type and location of microbial challenge.

## Introduction

Mucosa-associated invariant T cells are unconventional T cells operating on the border between the innate and adaptive immunity and respond promptly in an innate-like manner to antigens presented by the MHC class I-related (MR1) protein (1, 2). Human mucosa-associated invariant T (MAIT) cells express a semi-invariant T cell receptor (TCR) characterized by the uniform use of the Vα7.2 segment paired with Jα12, 20, or 33, whereas the β-chain diversity is broader but still limited (3–7).

The naturally occurring MR1-presented MAIT cell agonists identified to date are metabolites derived from the riboflavin biosynthesis pathway (8, 9). This limited set of antigens coupled with the high evolutionary conservation of MR1 (10) has favored the notion that MAIT cells may be functionally homogeneous and responding in a largely undifferentiated manner to microbes capable of riboflavin production. However, recent studies have demonstrated that MAIT cells are in fact fairly heterogeneous in their phenotype and function, and that their response patterns are influenced by a range of factors.

## Mait Cell Response Patterns Vary Based on Microbial Stimuli and Tissue Localization

We recently found that peripheral blood MAIT cells respond differently to distinct microbes in both the quality and quantity of cytokines produced (7). The Gram-negative bacterium *Escherichia coli* induced production of interferon (IFN)γ and tumor necrosis factor (TNF), as well as TCR downregulation, at significantly higher levels than the opportunistic fungus *Candida albicans*, and the MAIT cell polyfunctional cytokine profile significantly differed in response to these microbes (7). Moreover, several studies have provided evidence that MAIT cell effector functions vary based on tissue localization. Upon bacterial stimulation, peripheral blood and gastric MAIT cells produce IFNγ and TNF, and degranulate (2, 4, 11–15), whereas MAIT cells from the female genital mucosa display a bias toward production of interleukin (IL)-17 and IL-22 (16). Moreover, in response to *Mycobacterium tuberculosis* stimulation, MAIT cells from tuberculous pleural effusions display an enhanced capacity to produce IFNγ, IL-17F, and granzyme B than circulating MAIT cells (17). Upon phorbol myristate acetate and ionomycin stimulation, MAIT cells from the liver and adipose tissue produce more IL-17 and IL-10, respectively, than their peripheral blood counterparts (18, 19). Data from mouse models further support a role of MAIT cells in the control of type 1 diabetes *via* maintenance of gut integrity and control of anti-islet autoimmune responses (20), as well as of pulmonary infection by *Francisella tularensis* live vaccine strain (LVS) (21, 22). Overall, these findings suggest the existence of MAIT cell response patterns that vary with tissue localization and depend on the microbes encountered.

Antimicrobial immune responses are an outcome of the interplay between effector cells, antigen-presenting cells (APCs), and microbes. Recent findings have indicated that MAIT cells are phenotypically heterogeneous and comprise functionally distinct subsets (7). Thus, the functional compartmentalization of the MAIT cell population, together with distinct characteristics of APCs and microbes, may influence MAIT cell responses upon microbial encounter.

## Mait Cells—not as Homogeneous as they First may Seem

Adult peripheral blood MAIT cells were long considered phenotypically homogeneous in that they express a restricted semi-invariant TCR α-chain and predominantly exhibit a CD45RO^+^CCR7^−^CD62L^−^CD28^+^ effector memory phenotype (3, 7, 23, 24), as determined by individual assessment of surface receptors (23, 24) and by screening of their surface immune-proteome (7). However, MAIT cells vary in their expression of TCR Vβ segments (3–7), and of the natural killer (NK) cell-associated receptor CD56 (7). Thus, the discovery of these phenotypically distinct MAIT cell populations suggested the existence of subsets that could potentially exhibit different functional properties.

## The TCR β-Chain Composition Influences Mait Cell Antimicrobial Responses

Although less diverse than that of other T cells (5, 6), the Vβ usage of MAIT cells adds some diversity to their overall TCR β-chain repertoire. We observed that the Vβ segment expression can influence MAIT cell responses, as MAIT cells expressing Vβ8^+^, Vβ13.1^+^, and Vβ13.6^+^ were hyporesponsive to *E. coli*, and Vβ13.2^+^ MAIT cells were slightly hyperresponsive to *C. albicans* when compared with the total MAIT cell population (7). Lopez-Sagaseta et al. (25, 26) had previously reported different binding affinities between MAIT cell TCRs with different Vβ segments and MR1 in complex with a MAIT cell agonist. Thus, while the semi-invariant α-chain is indispensable for TCR recognition of MR1–ligand complexes (25, 27), the TCR β-chain may influence MAIT cell antimicrobial responses by fine-tuning the overall TCR–ligand–MR1 interaction. In light of the aforementioned findings, one can speculate that localization or accumulation of Vβ13.2^+^ MAIT cells, which comprise a significant proportion of the total MAIT cell population (7), at sites of *C. albicans* colonization, such as the genitourinary tract (28), could boost local immune responses against this opportunistic pathogen.

Mucosa-associated invariant T cell subpopulations defined by Vβ expression also have differential proliferative capacity *in vitro*. MAIT cells that express the more abundant Vβ’s proliferate more *in vitro* in response to *E. coli* than the less abundant ones (7). This finding raises the possibility that the *in vivo* interactions with microbes believed to drive the expansion of MAIT cells from the low frequencies seen in cord blood also shape the Vβ repertoire by selectively driving the expansion of more responsive MAIT cell subsets in an antigen-dependent manner. If this is the case, the MAIT cell TCR repertoire might be influenced by vaccination strategies that expose individuals to microbial antigens. In agreement with this, Howson et al. (29) recently reported a preferential expansion of certain MAIT cell clonotypes in human volunteers challenged with *Salmonella enterica* serovar Paratyphi A (29). Interestingly, the MAIT cell clonotypes that expanded *in vivo* were more strongly activated *in vitro* in an MR1-dependent manner than those that contracted during infection, potentially due to higher functional avidity between their TCRs and MR1 ligands (29). Thus, the MAIT cell TCR β-chain repertoire may function as a bacterial infection signature of any given individual. Furthermore, factors such as the geographic location, diet, or medication usage [all of them known to affect the microbiota (30, 31)] might shape the MAIT cell TCR β-chain repertoire as well. Hinks et al. (32) reported that the levels of MAIT cells in peripheral blood and bronchial tissues were affected in steroid-treated chronic obstructive pulmonary disease patients when compared with non-steroid-treated patients (32). However, it remains to be investigated if this or any other factor influences the MAIT cell TCR β-chain repertoire through its effect on the microbiota.

## CD56 Marks a Mait Cell Subset with Enhanced Innateness

A proportion of human MAIT cells in peripheral blood express the NK cell marker CD56 (7). Interestingly, the CD56-expressing MAIT cells have a higher capacity to respond to IL-12 and IL-18 than their negative counterparts (7). This can possibly be explained by their higher expression levels of IL-12R and IL-18R, as well as higher levels of the transcription factors PLZF, Eomes, and T-bet (7). The higher responsiveness of CD56^+^ MAIT cells to innate cytokines may make them more efficient in mounting MR1-independent responses during viral and bacterial infections, as well as sterile inflammatory conditions. In addition, CD56^+^ MAIT cells are reportedly more abundant in the liver than in peripheral blood (19, 33). Whether this MAIT cell subset has protective, pathogenic, or modulatory roles in liver diseases such as viral hepatitis remains to be determined.

**Figure 1:**
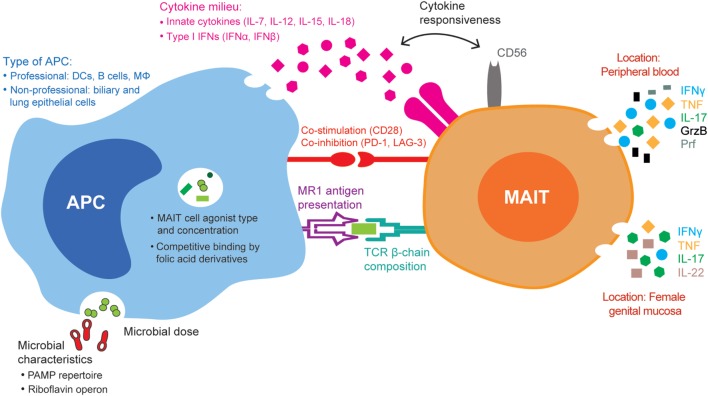
Summary of the mucosa-associated invariant T (MAIT) cell, antigen-presenting cell (APC), and microbial factors that may influence MAIT cell antimicrobial responses. The MAIT cell TCR β-chain and the expression of CD56 and of co-stimulatory and co-inhibitory receptors influence their responses to microbial stimuli. Microbes with distinct pathogen-associated molecular pattern (PAMP) repertoires and differential expression of the riboflavin operon differently activate APCs and influence the type and amount of MAIT cell agonists or folic acid derivative/non-stimulatory compounds that are presented to MAIT cells, respectively. APCs differ in the amount of MHC class I-related–antigen complexes presented to MAIT cells, the type and amount of innate cytokines produced, as well as the panel of co-inhibitory and co-activating molecules expressed. The combination of all these factors influences downstream MAIT cell responses, both cytokine production and cytolytic capacity, in different tissues, such as peripheral blood and the female genital mucosa.

In summary, the type and magnitude of effector functions mounted in response to stimuli can be influenced by factors intrinsic to the MAIT cells: the TCR β-chain composition and CD56 expression (Figure 1). Thus, the relative amounts of CD56^+^ and CD56^−^ MAIT cells and of Vβ-defined MAIT cell subsets, the latter potentially already determined by previous microbial encounters *in vivo*, might play an important role in determining how the MAIT cell compartment will respond to a new antigenic challenge.

## Differential Dependence on MR1 for Mait Cell Cytokine Production

At steady state, MR1 is mostly retained intracellularly (34–37) and traffics to the cell surface upon ligand availability and APC activation (34, 36, 38). Interestingly, blocking experiments have revealed differential MR1-dependency in MAIT cell responses (7). In responses to both *E. coli* and *C. albicans*, most IFNγ and virtually all TNF produced by MAIT cells was dependent on the TCR–MR1 interaction (7). However, a small proportion of IFNγ was produced in an MR1-independent manner (7). The dependency on MR1-mediated antigen presentation for MAIT cell production of TNF suggests tight regulation of their pro-inflammatory responses. This may be an important regulatory mechanism to prevent MAIT cell activation in response to riboflavin biosynthesis-competent commensal microbes that do not actively produce MAIT cell agonists at steady state, but still reside close to MAIT cells. Moreover, the low levels of MR1 on most cells at steady state may prevent continuous activation of MAIT cells by MR1 ligands from commensal microbes. As MR1 can bind extracellular ligands directly on the cell surface, these ligands, even if present at homeostatic levels, could otherwise lead to unnecessary MAIT cell pro-inflammatory responses (36).

## Innate Cytokines Activate Mait Cells in an MR1-Independent Manner

Microbes activate APCs to secrete cytokines, such as IL-12 and IL-18, which induce IFNγ production by MAIT cells independently of TCR signaling and MR1 (39). In agreement with these initial findings, Jesteadt et al. (40) recently reported different MAIT cell responses to two microbes that differ in their capacity to activate the inflammasome, a proinflammatory innate immune system collection of receptors and sensors that involve activation of caspase-1 and inflammatory molecules to both microbes and host-derived proteins (40, 41). In contrast to *Francisella tularensis* LVS, *Francisella novicida* is a strong inflammasome activator that induced high levels of IL-18 production by macrophages and subsequent high levels of IFNγ production by murine MAIT cells (40). Moreover, the magnitude of the *in vitro* IFNγ production in response to *F. novicida* was directly influenced by the concentration of IL-18 in the cultures (40). Therefore, one can expect differential MAIT cell responses to microbes with different ability to induce IL-18 production by the APCs.

Other cytokines can have a range of effects on peripheral blood MAIT cells. In the absence of microbial stimulation, IL-7 induces GrzB and upregulates Prf expression without concomitant production of cytokines (14), whereas IL-15 in combination with IL-18 and/or IL-12 induces IFNγ and GrzB production (42–44). Upon stimulation with suboptimal doses of *E. coli*, both IL-7 and IL-15 augment cytokine and cytolytic molecule expression by MAIT cells (14, 42). Moreover, the combination of MR1 antigen presentation with either IL-12, or IL-7 and IL-12, induces GrzB production by MAIT cells in response to *E. coli* (13) or nontypeable *Haemophilus influenzae* (NHTi), respectively (45). Thus, it is plausible that APCs shape MAIT cell antimicrobial responses through the cytokines they produce upon microbial-mediated activation. In fact, the capacity of MAIT cells to be activated by cytokines alone underlies their ability to respond *in vitro* to several viruses, including dengue virus, influenza virus, and hepatitis C virus, in a process dependent on IL-12 and IL-18, IL-18 alone, and IL-18 and IL-15, respectively (43, 46). In addition, both IFNα and IFNβ were shown to activate MAIT cells (43, 47) and further contribute to the MAIT cell response to HCV (43). Moreover, MAIT cells can respond to the superantigen staphylococcal enterotoxin B independently of MR1 and in an IL-12, IL-18, TCR Vβ, and HLA class II-dependent manner (48, 49).

## Different Types of APCS Vary in Key Functions Required for Mait Cell Activation

As the *MR1* gene is ubiquitously expressed (50–52), many different cell types are able to present antigen to MAIT cells. The repertoire of innate cytokines and the extent to which MR1 is upregulated and brought to the cell surface upon activation and ligand availability vary not only with the type of stimulation but also with the type of APC (38, 53). Professional APCs (DCs, macrophages, and B cells) are efficient in microbe internalization and processing, as well as in delivering co-stimulatory signals to T cells. Kurioka et al. showed that the MR1-dependency of the MAIT cell response to pneumococci varied with the type of APC used (54). While the MAIT cell response in the presence of monocytes was MR1-independent, it was partially MR1-dependent in the presence of monocyte-derived macrophages (54). We previously observed that the addition of anti-CD28 to *E. coli*-fed monocytes cultured with Vα7.2^+^ cells boosted MAIT cell IFNγ production (55), thus indicating that monocytes are not intrinsically efficient in delivering co-stimulatory signals and that the magnitude of the MAIT cell response varies with the degree of co-stimulation provided.

In conclusion, several aspects of the APC shape the magnitude and quality of MAIT cell antimicrobial responses. Such APC-intrinsic factors include the surface expression of MR1–antigen complexes, the innate cytokines produced, and the panel of co-stimulatory and co-inhibitory receptors expressed upon microbial exposure (Figure 1). MAIT cells are likely to encounter different APCs *in vivo*, and their responses will ultimately be influenced by the type and representation of APCs.

## Microbe Growth Conditions Influence the Production of MR1-Presented Ligands

Mucosa-associated invariant T cells sense microbes through antigens presented by MR1 on the surface of APCs. The naturally occurring activating antigens identified thus far belong to the riboflavin biosynthesis pathway (8, 9), expressed in many different species of bacteria and fungi (56, 57). However, the ability of a microbe to activate MAIT cells depends not only on its capacity to produce MR1-presented agonists but also on whether MAIT cell non-stimulatory MR1-binding compounds are also produced and to what extent.

The type and concentration of MR1-presented compounds vary with the microbial growth conditions. 5-(2-oxopropylideneamino)-6-d-ribitylaminouracil (5-OP-RU), the most potent MAIT cell agonist identified thus far, requires the riboflavin intermediate precursor 5-amino-6-d-ribitylaminouracil (5-A-RU) and either methylglyoxal or glyoxal for its generation (9), whereas natural MAIT cell non-stimulatory compounds derive from folic acid (8, 58, 59). The concentration of these precursor molecules at effector sites will likely dictate the amount of antigenic and non-antigenic MR1-presented compounds. It was recently shown that *Streptococcus pneumoniae* clinical isolates respond to exogenous availability of riboflavin by downregulating the *ribD* gene (which encodes the enzyme pyrimidine deaminase/reductase essential for the production of 5-A-RU), with a consequent decrease in MAIT cell stimulatory potential (60). On the other hand, heat-stress was shown to induce the riboflavin operon in pneumococci, with upregulation of the riboflavin pathway genes within 2–4 h under such conditions (54).

It is also possible that other, yet unknown MAIT cell antigens with different requisites for their formation exist. Meermeier et al. (61) reported that MR1-restricted non-classical TRAV1-2^-^ (Vα7.2^-^) MAIT cells could be activated in an MR1-dependent manner by *Streptococcus pyogenes*, a riboflavin biosynthesis-incompetent microbe. This suggests that MR1 can present MAIT cell agonists other than riboflavin metabolites (61).

Other microbial products that are not presented by MR1 may also influence MAIT cell responses. For instance, lactate was shown to dampen NK and T cell activation in response to *Staphylococcus aureus* (62). Furthermore, short-chain fatty acids derived from bacterial fermentation, such as acetate, butyrate, and propionate, promote differentiation of T cells in a cytokine milieu-dependent manner (63, 64). Further investigation is required to determine if MAIT cells respond similarly to these microbial products.

In conclusion, the type of MR1-presented compounds and other bioactive microbial products will likely shape the functional characteristics of MAIT cell antimicrobial responses, as previously exemplified in *in vitro* competition experiments between MAIT cell activating and non-activating MR1-binding compounds (58, 59, 65).

## Microbial Genetic Background may Play a Role in Mait Cell Responses

Recent studies showed that different *S. pneumoniae* isolates activated MAIT cells to different extents, as assessed by CD69 upregulation and IFNγ production (54, 60). Interestingly, Hartmann et al. found that *S. pneumoniae* isolates with similar MAIT cell activating properties grouped together with regard to their multilocus sequence type, suggesting a link between the MAIT cell response and microbial genetic background (60). The pneumococcus strain groups inducing higher levels of MAIT cell responses expressed significantly higher levels of the *ribD* gene and of MAIT cell ligands (60). Thus, differences in the genetic background between microbes influence their capacity to activate MAIT cells.

## The Microbial PAMP Signature and Propensity for Phagocytosis can Affect Mait Cell Responses

Different classes of microbes express distinct PAMPs, which can trigger toll-like receptors (TLRs) in APCs. Recently, Ussher et al. showed that the IFNγ production by MAIT cells upon *E. coli* stimulation can be positively or negatively affected by pretreatment of APCs with TLR agonists (38). Therefore, the PAMP–TLR interaction might be another factor shaping MAIT cell antimicrobial responses.

Given that phagocytosis of particles depends on their size and shape (66), geometrically different microbes may have different propensity to be phagocytosed. Moreover, certain microbes contain a polysaccharide capsule, and variations in this structure are known to influence the rate of phagocytosis (67, 68). Thus, the intracellular microbial load may vary quite extensively with the type of microbe. Interestingly, by using *S. enterica* serovar Typhi and *E. coli* as microbes and a B cell line as APCs, Salerno-Goncalves et al. found that the quality of the MAIT cell response depended on the microbial load (69).

In summary, MAIT cell antimicrobial responses can be influenced by several microbe-intrinsic factors, including their genetic background, physical characteristics, and PAMP repertoire, as well as their ability to produce MAIT cell antigens and other microbial products (Figure 1). These factors will not only influence MAIT cell functions but also dictate the amount of microbe that is required for optimal responses. In our study of MAIT cell responses to *E. coli* and *C. albicans*, we found that the optimal dose necessary for maximal MAIT cell activation, as assessed by CD69 upregulation and IFNγ production, was much higher for *E. coli* than for *C. albicans* (7).

## Concluding Remarks

In conclusion, numerous factors influence the quality and magnitude of MAIT cell antimicrobial responses, including the MAIT cell TCR β-chain composition, the expression of NK cell-associated receptors, and the TCR–ligand–MR1 interaction. Predominance of MAIT cell subsets with distinct effector functions at sites of microbial invasion and their co-localization with functionally heterogeneous conventional CD4^+^ and CD8^+^ T cells that recognize distinct antigens (70–72) builds multifaceted immune barriers of immunosurveillance able to efficiently target pathogens with different requirements for eradication.

## Author Contributions

JD, CB, EL, and JS wrote the manuscript; MS provided the figure; MS, KL, JE, MB, TP, and J-BG contributed to manuscript editing and revision; JD and JS revised the final manuscript.

## Conflict of Interest Statement

The authors declare that the research was conducted in the absence of any commercial or financial relationships that could be construed as a potential conflict of interest.
